# Intra-articular angiolipoma of the knee: a case report

**DOI:** 10.1186/1758-2555-2-10

**Published:** 2010-04-13

**Authors:** Makoto Nishimori, Masataka Deie, Nobuo Adachi, Atsushi Kanaya, Atsuo Nakamae, Mitsuo Ochi

**Affiliations:** 1 Department of Orthopedic Surgery, Programs for Applied Biomedicine, Division of Clinical Medical Science, Graduate School of Biomedical Sciences, Hiroshima University, 1-2-3 Kasumi, Minami-ku, Hiroshima 734-8551, Japan

## Abstract

We report a case of intra-articular angiolipoma of the knee. This case report describes our experience in excising an intra-articular angiolipoma of the knee joint. Complete resection under arthroscopy was performed in a 30-year-old man. Two years after the surgery, no evidence of recurrence was seen. Intra-articular angiolipomas should be considered in the differential diagnosis of intra-articular masses in adolescents with recurrent hemarthrosis without trauma.

## Background

Angiolipomas are uncommon benign soft-tissue tumors that typically arise in the subcutaneous tissues of young patients [1]. The forearm is decidedly the most common location, with almost two thirds of all angiolipomas found here [2]. A recent review of the literature revealed several reports of these tumors occurring in locations other than subcutaneous regions [3-5].

Intra-articular angiolipoma of the knee is a very rare cause of knee pain, hemarthrosis and dysfunction. To our knowledge, there has been no reported case of intra-articular angiolipoma of the knee in English. We report on this exceedingly rare case and review the previous angiolipoma reports.

## Case report

**Figure 1:**
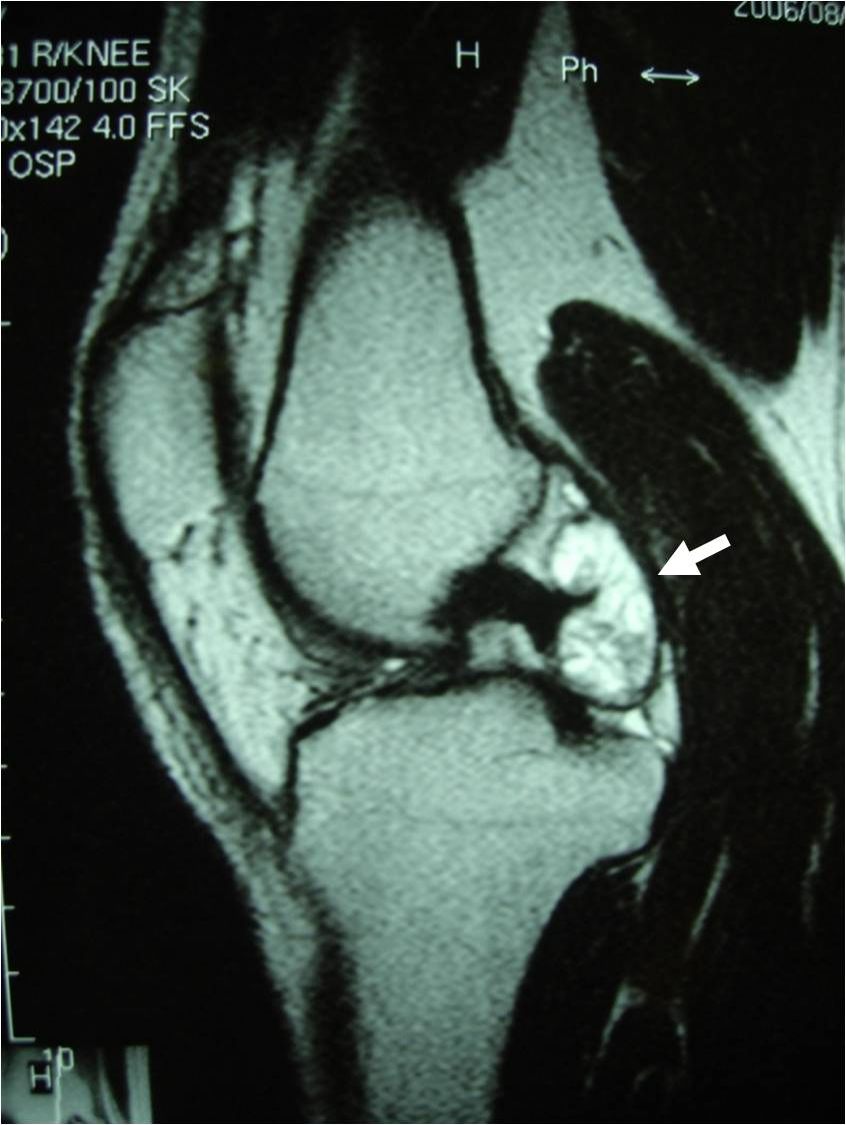
**Preoprerative MRI**. Magnetic resonance imaging of the right knee showed a 2.5 cm tumor located in the dorsal area of the posterior cruciate ligament (arrow). The tumor exhibited a fluctuating high and low signal intensity with low signal septa around the tumor on T2-weighted images.

A 30-year-old man presented to our clinic complaining of a recurrent swollen and painful knee over a period of 6 months without significant trauma. Examination revealed swelling and ballottement of the patella in the right knee joint, and aspiration of bloody joint fluid suggested a hemorrhage. The range of motion of the knee was limited to between 0 and 130 degrees with terminal flexion pain. There was no point of tenderness and instability of the knee. A plain x-ray showed no atrophic or degenerative change of the knee. Magnetic resonance imaging (MRI) showed a 2.5 cm polylobulated tumor localized in the dorsal area of the posterior cruciate ligament (PCL). MRI of the tumor exhibited a slightly high signal intensity, in contrast to muscle on T1-weighted images, a fluctuating high and low signal intensity with low signal septa around the tumor on T2-weighted images and an inhomogeneous signal, enhanced moderately on T1-weighted images with gadolinium (Gd)-enhancement (Fig 1).

**Figure 2:**
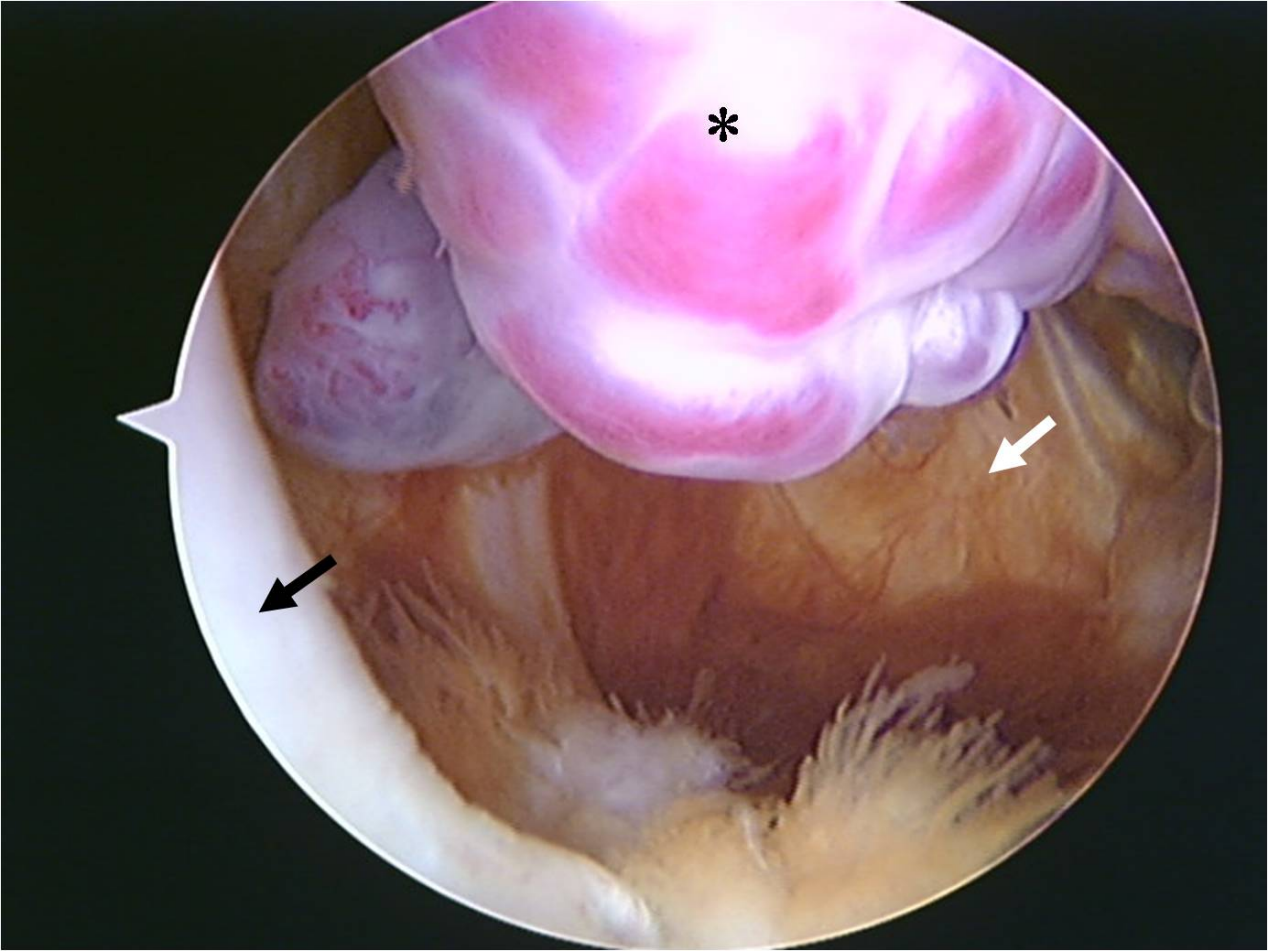
**Arthroscopic view of the knee**. Arthroscopic view from infra-medial portal of the tumor (asterisk), situated between the posterior cruciate ligament (black arrow) and the posterior portion of the medial femoral condyle. The appearance of the polypoidal nodule resembled a bunch of grapes. (White arrow indicates capsule.)

**Figure 3:**
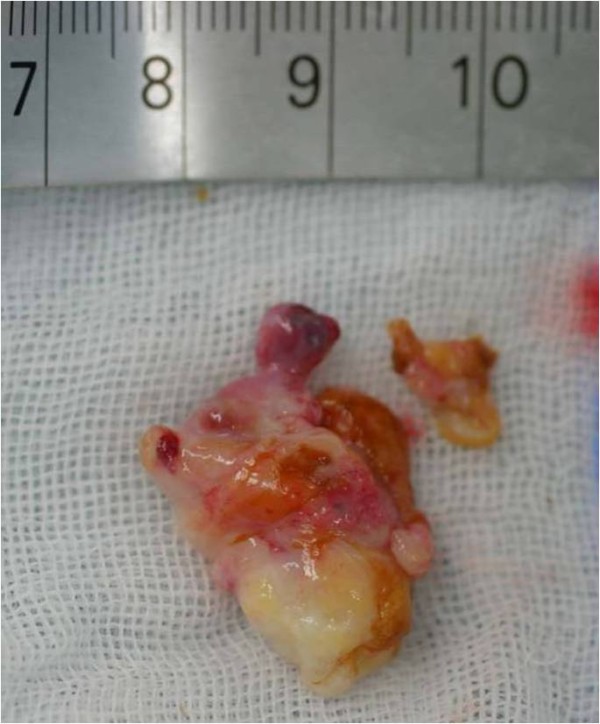
**Gross appearance of the resected specimen**. The macroscopic appearance of the resected tumor. The tumor consists of yellowish and reddish polylobular tissue.

**Figure 4:**
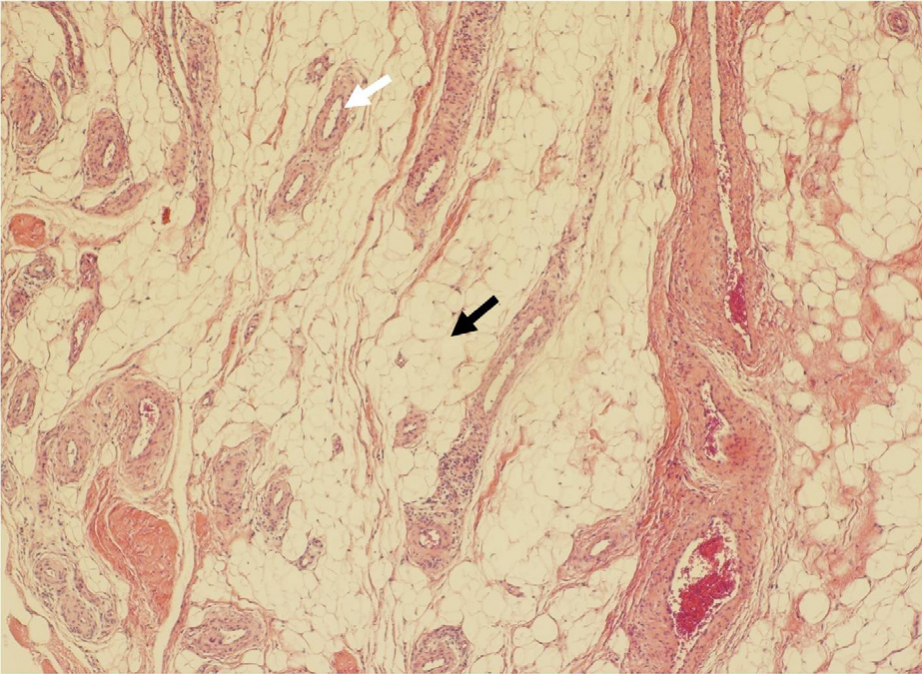
**Histologic appearance of the tumor**. Histological appearance of an intra-articular angiolipoma. The tumor consists of mature adipose cells (black arrow) mixed with complicated and aggregated capillaries (white arrow). (Hematoxylin and eosin, original magnification ×40)

Arthroscopy revealed a polypoidal nodule measuring 20 × 20 × 10 mm and resembling a bunch of grapes, in the posterior portion of the PCL. The macroscopic appearance of the mass was consistent with synovial hemangioma and lipoma (Fig 2). A complete excision of the mass was easily performed using standard infra-patellar arthroscopic portals with an additional posteromedial portal (Fig 3). Histological examination of the excised mass tissue revealed mature adipose cells mixed with complicated and aggregated capillaries consistent with a diagnosis of angiolipoma (Fig 4).

After arthroscopic excision, the patient remained asymptomatic at the 24-month follow-up, and repeat MRI scans of the knee showed no evidence of recurrence.

## Discussion

Angiolipomas were first reported by Bowen in 1912 [6], and in 1960 Howard et al. [7] were the first to establish angiolipomas as a new entity by reporting 248 cases. Angiolipomas are considered to be vascular lipomas, and they account for 5% to 17% of all lipomas [1,7]. To the best of our knowledge, this is the first case report in English on intra-articular angiolipomas of the knee. Gonzalez-Crussi et al. [8] proposed classifying angiolipomas into infiltrating and non-infiltrating types, but lesions described as being deep infiltrating angiolipomas have now been recognized by the WHO as being intramuscular hemangiomas [9].

The etiology of angiolipomas remains unclear, and the relationship between lipomas, angiolipomas, angiomyolipomas and haemangiomas needs to be further elucidated. Most authors accept that these different lesions may arise from the abnormal development of a primitive pluripotential mesenchymal cell from which adipose tissue, smooth muscle and vascular endothelium develop [3].

Histologically, an angiolipoma consists of two predominant tissue elements, adipocytes and interspersed vascular structures. The lesion is always encapsulated by subcutaneous yellow nodules with reddish areas corresponding to vessels. The vessels are small-caliber capillaries and often contain fibrin thrombi. This is a characteristic feature of this lesion, with surrounding mature fat [2,10]. For differential diagnoses, the distinction in part depends on the density of the vessels. In the case of a hypo vascular lesion, it may be difficult to distinguish it from a lipoma, but the existence of fibrin thrombi may help with this distinction. In the case of a hyper vascular lesion, one possible differential diagnosis is a hemangioma. It might be difficult to distinguish it from a hemangioma, but there is no lipomatous component in a hemangioma, which will help to make the correct diagnosis.

Our patient had recurrent intra-articular hemarthrosis without significant trauma. Clinically, recurrent intra-articular hemorrhage without trauma is defined by a synovial hemangioma, pigmented villonodular synovitis (PVS), idiopathic hemorrhage and hemophilic arthropathy. The differential diagnosis, of synovial hemangioma or localized PVS for intra-articular bleeding could be reached after detecting an intra-articular tumor-like mass on MRI.

Regarding the differential diagnosis of these entities on MRI, the characteristics of synovial hemangioma on MRI are that iso signal intensity contrasted to muscle on T1-weighted images, a strong high signal intensity with low signal intensity septa on T2-weighted images and inhomogeneous enhancement of internal lesions on T1-weighted images with Gd-enhancement [2,11]. In contrast, the characteristics of PVS on MRI are that an iso-intense or low signal intensity compared with muscle on T1-weighted and T2-weighted images and inhomogeneous enhancement on T1-weighted images with Gd-enhancement [2,12].

In this case, the tumor exhibited slightly high signal intensity in contrast to muscle with low intensity area on T1-weighted images, a fluctuating high and low signal intensity with low signal septa around the tumor on T2-weighted images and inhomogeneous enhancement on T1-weighted images with Gd-enhancement. From differences of these characteristics on MRI, we might be able to distinguish this case from the other tumors. However, MRI is useful, albeit imperfect, in distinguishing these tumors with accuracy. Therefore, diagnosis could be made arthroscopically and accurately with histopathological evaluation.

Surgical excision is curative, and there is no evidence of malignant transformation [2]. We depicted the mass lesion located posterior to the PCL. As we made a posteromedial portal which we usually use for PCL reconstruction[13], we could excise the tumor safely under arthroscopy. However, if it is impossible to excise the tumor under arthroscopy, the approach to the tumor must be unequivocally changed, from arthroscopic resection to arthrotomy.

In conclusion, an intra-articular angiolipoma of the knee can be the cause of knee pain and recurrent hemarthrosis. Diagnosis can be made arthroscopically, but histopathological evaluation is more accurate. Excision should be performed either arthroscopically if the tumor is completely visible, or using arthrotomy.

## Consent

Written informed consent was obtained from the patient for publication of this case report and any accompanying images. A copy of the written consent is available for review by the Editor-in-Chief of this journal.

## Competing interests

The authors declare that they have no competing interests.

## Authors' contributions

MO, MD, NA and MN carried out the surgical treatment and MD, AK and MN contributed to the follow-up examinations in an outpatient clinic. MD, MN and AN co-wrote the paper, discussed the results and commented on the manuscript. All authors have read and approved the final manuscript.
